# Protecting the gains of malaria elimination in China

**DOI:** 10.1186/s40249-020-00661-y

**Published:** 2020-04-24

**Authors:** Xinyu Feng, Joshua Levens, Xiao-Nong Zhou

**Affiliations:** 1 National Institute of Parasitic Diseases, Chinese Center for Diseases Control and Prevention; Chinese Center for Tropical Diseases Research, Shanghai, 200025 China; 2 WHO Collaborating Centre for Tropical Diseases; National Center for International Research on Tropical Diseases, Ministry of Science and Technology; Key Laboratory of Parasite and Vector Biology, Ministry of Health, Shanghai, 200025 China; 3 RBM Partnership to End Malaria, Geneva, Switzerland

## Background

About half of the world’s population risks contracting malaria, which despite the effective treatments available globally, remains a life-threatening affliction, particularly for the poorest and most vulnerable populations. To raise awareness, advocacy, and action towards defeating the disease, which was responsible for approximately 228 million debilitating infections and 405 000 deaths at last count in 2018, the international community has, since 2008, commemorated World Malaria Day on 25 April. Events all over the world have provided opportunities for malaria affected countries to learn from each other’s experiences and support each other’s efforts. Likewise, it has been an occasion for development partners across all countries to showcase their efforts against malaria, reflect on successes, and assess the challenges ahead. This year the World Malaria Day takes its theme from the pan-African campaign, first launched in Senegal, known as “Zero palu! Je m’engage” or “Zero Malaria Starts with Me”.

“Zero Malaria Starts with Me” campaign highlights the importance of personal responsibility in addressing the disease and the different roles that everyone can play, from their workplaces to their homes. This theme of personal responsibility in fighting against a global epidemic is especially resonant this year as countries respond to the unprecedented challenges of the COVID-19 pandemic. As with COVID-19, acting against malaria requires strong disease surveillance systems, reporting, community response, and treatment follow up.

As China prepares to commemorate the upcoming World Malaria Day, the links between the malaria and COVID-19 response will be featured by the special theme on “*Eliminating malaria and containing COVID-19: co-mitigation of imported cases and re-establishment*”. Addressing both malaria and COVID-19 requires a disease surveillance-reponse system for febrile cases that includes: (i) early detection, (ii) early reporting, (iii) early diagnostic confirmation, and (iv) early treatment. This system for febrile patients can both support China in its efforts to prevent indigenous malaria transmission and contain COVID-19 infections at the same time.

### Prevention of malaria retransmission by monitoring the imported cases at a country level

While China has made tremendous progress in its national malaria elimination programme since 2010, a great deal of work remains to consolidate the achievement of zero indigenous malaria cases, particularly with respect to surveillance. Malaria cases from abroad are detected in China on an almost daily basis. Moreover, the malaria vector mosquitos still thrive with the potential to transmit any imported cases that may be missed. This situation is additionally challenged by the complicated ecological settings along the Yunnan border areas, neighboring three malaria endemic countries: Lao People’s Democratic Republic, Myanmar, and Vietnam [1–3], as well as the importation of cases from Chinese workers returning from outside the region, such as from Africa. Estimates of these imported cases exceed over 2500 per year and have impacted almost all provinces of China over the past decade. China must therefore maintain the continuity of its febrile surveillance-response system, with adequate numbers of professional staff, sustained investments in prevention and treatment, and ongoing policy support to effectively prevent the resurgence of local infections and transmission. To ensure that all imported cases are closely monitored and promptly addressed, we make the following recommendations:

1. **Strengthen political commitment and leadership, and sustain investments**

Political commitment and leadership have been essential for China’s achievements to date. However, after reaching the zero malaria milestone, there is a risk of turning attention away from the necessary actions and investments in surveillance and infection control to prevent onward malaria transmission in China. Strengthening the political commitment and leadership, and sustaining investments in the surveillance response system is therefore critically important. A total of US$ 116 million was spent on malaria control and elimination in Chinese programme on malaria control from 2002 to 2012 with financial resources from the Global Fund to Fight AIDS, Tuberculosis and Malaria. No longer able to depend on exernal financing for malaria elimination efforts after 2012, because the central government of China have invested an estimated US$10 million each year in the national malaria elimination programme, Chinese funding commitments and the political leadership to realize them are more important than ever.

2) **Establishment of the long-term mechanism for managing imported malaria cases and mobile population**

With the advancement of global integration, the number of imported cases has been rising. At present, there are many weak points in the prevention and treatment of imported malaria cases. First, because the management of imported malaria involves many departments, effective management mechanisms have not been further improved, and as such, various management measures need to be strengthened in implementation of malaria elimination programmes at all levels [4, 5]. Second, the mobile population may lack the awareness, knowledge, and conditions of malaria protection, active consultation and standardized treatment [6]. Without education about the disease, workers risk infection while traveling in highly endemic countries abroad. In addition, the lack of education can act as a barrier for seeking timely treatment with appropriate medicines. Therefore, the proper management of imported malaria cases and population movement is critical to prevent outbreaks and the reintroduction of malaria in the future.

3) **Maintenance of post-malaria elimination surveillance and response capacity**

China has built a nationwide network for reporting epidemics and a comprehensive monitoring system with appropriate indicators. However, the significant decline in malaria cases has meant that professionals engaged in malaria prevention and treatment have become deficient in their skills and practice. In some areas, there are no departments specializing in malaria prevention and treatment. Building and sustaining the capacity of the disease control institutions and construction of a talented team remains challenging. The relevant applied research and development of early and sensitive diagnostic tools to discover and dispose of local active outbreaks and respond to unexpected imported malaria cases also urgently improved. In order to sustain the goal of eliminating malaria, capacity building needs to be a top priority [7, 8].

4) **Improving information communication and multi-sectoral cooperation**

Ending malaria and preventing its reintroduction requires not only the health sector, but engagement and cooperation across multiple sectors [9]. Ministries, departments, and agencies which deal with the flow of people and goods such as commerce and tourism can effectively cooperate with health officials to perform joint disease surveillance and control work to prevent secondary transmission caused by imported malaria [1, 7]. Sectors which address the environment, agriculture, housing, energy and water can support malaria vector control efforts. Moreover, across all sectors, close cooperation and effective communication can help to raise awareness of malaria infection risk, appropriate diagnosis and treatment, and promote advocacy efforts to sustain investments in surveillance-response systems for management of febrile patients.

### Improving management of migrated population at a global level

To date, 38 countries and territories having been certified malaria-free by the WHO. China is poised to join their ranks soon. This may very well become not only a milestone in the history of disease prevention for China, but a critical achievement for global malaria eradication. Elimination of malaria in China has been supported by substantial achievements in health and development more broadly, in addition to the malaria-specific investments and interventions. We recognize therefore the importance of considering malaria elimination not only within the broader framework of responding to other epidemics such as COVID-19, but also as part of the Sustainable Development Goals across all sectors as part of a whole-of-government approach. If successful in achieving malaria-free certification by the WHO, China’s surveillance-reponse system should continue to focus on people who return from malaria-endemic areas of Africa and Southeast Asia, strengthening the disease surveillance-response systems to prevent the introduction of imported malaria cases or foci, and maintaining malaria surveillance-response capabilities both in the laboratory and epidemiological workforce. Finally, based on China’s experiences, we share the following experiences for the global malaria response:

1. Urgent action is needed to get the global malaria response back on track. Collective political will, continued multisectoral investments and a unified and coordinated effort are needed. National level malaria programmes need high-level and broad based support to implement ambitious strategic plans.
2. Scale up of effective prevention, diagnosis, and treatment interventions needs to be augmented by introduction of new and more effective technologies where appropriate, including next generation insecticides in long-lasting insecticidal nets and indoor residual spraying to manage insecticide resistance. In addition, there are valuable opportunities for exploring the application of new tools for surveillance and response, including through remote sensing and artificial intelligence, among others.
3. Strengthening regional and international cooperation is absolutely crucial to regional malaria elimination and global eradication. This will include sharing disease surveillance and response data, sharing experiences among programmes, accelerating the provision of global public goods, and providing sustainable financing to reach the end of malaria.

## Conclusion

As we commemorate World Malaria Day 2020 against the backdrop of the COVID-19 response, we are reminded as well that the systems, competencies, and strategies that support malaria elimination can also play an important role in addressing epidemics, febrile illnesses more generally and contribute to global health security. It is important that we remember the global malaria elimination agenda not as a distraction from the COVID-19 crisis, but as a critical enabler of the effort.

## Data Availability

Not applicable.
